# Resurgent and delayed malaria

**DOI:** 10.1186/s12936-022-04098-6

**Published:** 2022-03-09

**Authors:** Brian Greenwood, Issaka Zongo, Alassane Dicko, Daniel Chandramohan, Robert W. Snow, Christian Ockenhouse

**Affiliations:** 1 Faculty of Infectious and Tropical Diseases, London School of Hygiene and Tropical Medicine, Keppel St, London, WC1E 7HT UK; 2 Institut de Recherche en Science de La Santé, Bobo-Dioulasso, Burkina Faso; 3 Malaria Research and Training Centre, University of Sciences Techniques and Technologies, Bamako, Mali; 4 Kenya Medical Research Institute-Wellcome Trust Collaborative Programme, Nairobi, Kenya; 5 Centre for Tropical Medicine and Global Health, Nuffield Department of Clinical Medicine, University of Oxford, Oxford, UK; 6 PATH – Malaria Vaccine Initiative, Washington, DC USA

**Keywords:** Malaria, Immunity, Rebound malaria, Resurgent malaria, Delayed malaria

## Abstract

The populations of moderate or highly malaria endemic areas gradually acquire some immunity to malaria as a result of repeated exposure to the infection. When this exposure is reduced as a result of effective malaria control measures, subjects who benefitted from the intervention may consequently be at increased risk of malaria if the intervention is withdrawn, especially if this is done abruptly, and an effective malaria vector remains. There have been many examples of this occurring in the past, a phenomenon often termed ‘rebound malaria’, with the incidence of malaria rebounding to the level present before the intervention was introduced. Because the main clinical burden of malaria in areas with a high level of malaria transmission is in young children, malaria control efforts have, in recent decades, focussed on this group, with substantial success being obtained with interventions such as insecticide treated mosquito nets, chemoprevention and, most recently, malaria vaccines. These are interventions whose administration may not be sustained. This has led to concerns that in these circumstances, the overall burden of malaria in children may not be reduced but just delayed, with the main period of risk being in the period shortly after the intervention is no longer given. Although dependent on the same underlying process as classical ‘resurgent’ malaria, it may be helpful to differentiate the two conditions, describing the later as ‘delayed malaria’. In this paper, some of the evidence that delayed malaria occurs is discussed and potential measures for reducing its impact are suggested.

## Background

Repeated exposure to *Plasmodium falciparum* leads to the acquisition of some protective immunity to this infection. Protection is acquired first against severe disease, then against uncomplicated clinical attacks of malaria and finally against malaria infection, although the latter is rarely complete. Consequently, when a highly effective malaria control intervention is introduced into a population for a limited period of time and then withdrawn, there is a risk that in the subsequent period the population which received the intervention may be at greater risk from malaria than if they had not received the intervention. This phenomenon is commonly termed ‘rebound malaria’.

In this paper, it is suggested that it may be helpful to differentiate two related but different epidemiological situations often considered under this heading. In the first situation, an intervention is applied to a whole population, or to a large part of a population, for a period of time and then withdrawn abruptly without there being a major and sustained reduction in the population of vector mosquitoes. This may result in an increase in the incidence of malaria to the level that was present before the intervention, i.e. a rebound to the previous level of infection, an event often termed ‘resurgent malaria’ in some of the early studies in which this phenomenon was described [1].

In the second situation, young children are protected from malaria with an effective intervention from early in life but the intervention is withdrawn when they reach a defined age. In this case, children may be more at risk of malaria in the years after the intervention is withdrawn than if they had not received the intervention, with the peak incidence of malaria moving to an older age than would otherwise have been the case. In this situation, the overall burden of malaria in these children may not have been prevented but just delayed. Although both of these events are due to lack of acquisition of naturally acquired immunity as a result of application of an effective intervention, it is suggested that it is helpful from the practical point of view to consider ‘resurgent malaria’ and ‘delayed malaria’ separately. The potential for the occurrence of ‘delayed malaria’ in young children is likely to be much greater than that of resurgence following interventions affecting a whole community, many of whose adult members will have developed strong immunity prior to the intervention which may still be adequate to protect them during the period after the intervention is withdrawn.

## Resurgent malaria

The classical study undertaken at Garki, northern Nigeria showed that it was possible to achieve a high level of malaria control using indoor residual spraying (IRS) combined with chemoprevention, even in an area with a very high level of malaria transmission, but that after the interventions were withdrawn, the incidence of malaria returned rapidly to the pre-intervention incidence [1]. Resurgence in malaria of this kind has been reported in many other countries when an effective antimalarial programme has been terminated prematurely without reducing malaria transmission substantially, as described in the comprehensive review by Cohen et al. [2]. For example, a recent study undertaken in Uganda, showed a rapid resurgence in malaria following termination of a successful malaria control programme that employed IRS and insecticide-treated nets (ITNs) [3] with efficacy restored once IRS was reintroduced [4]. In some cases, resurgence has led to an epidemic, as was seen in Sri Lanka on termination of the country’s first malaria eradication programme [5]. However, a resurgence in morbidity and mortality from malaria is not inevitable if there has been substantial progress in control of the key malaria vectors, leading to a reduction in transmission, or greatly improved access to diagnosis and effective treatment, during the period in which effective control was achieved and these measures are sustained.

## Delayed malaria

Concern that administration of effective malaria control interventions to young children living in highly endemic areas that was not sustained into later life would lead to severe malaria in older children was one of the reasons that for many years led the World Health Organization (WHO), and other international organizations, to take an unfavourable stance on the use of chemopreventive measures in young children living in a malaria endemic area. However, this view has changed with chemoprevention in the early years of life, given for a limited period, now being recommended in the form of Intermittent Preventive Treatment in Infants (IPTi) and Seasonal Malaria Chemoprevention (SMC) in older children. Consequently, the strongest evidence that ‘delayed malaria’ may occur comes from studies of chemoprevention.

An early study of seasonal chemoprophylaxis undertaken in Gambian children evaluated the incidence of uncomplicated malaria in young children who had received seasonal chemoprevention for 1 to 5 years. During the period of the intervention, there was a marked and sustained reduction in mortality and morbidity from malaria [6]. However, in the year after the intervention was stopped, when children had reached five years of age, an increase in the incidence of uncomplicated clinical malaria was seen which was most marked in children who had received chemoprevention from the age of three months to five years [7]. Hospital admissions with severe malaria were not recorded in this study, but there was no increase in deaths in the five years after the intervention, although the number of events was too small to have excluded a small effect [7]. In two more recent studies of SMC, previously called intermittent preventive treatment in children (IPTc), undertaken in Burkina Faso and Mali, an increase was seen in the incidence of uncomplicated malaria in the year after one year of intervention in both countries but this was only modest (IRR 1.12 [95% CI 1.04, 1.20] in Burkina Faso, and 1.09 [95% CI 0.99, 1.21] in Mali, respectively [8, 9]. However, in all three studies of seasonal chemoprevention, the reduction in cases during the period in which the intervention was given far exceeded the increase in cases in the subsequent follow-up period. SMC is now being deployed widely across countries of the Sahel and sub-Sahel but there has been no formal, published study of the risk of ‘delayed malaria’ in children who have received annual SMC from the age of three months to five or ten years of age when the intervention is no longer given. In order to address this issue, the authors are currently undertaking a study in Burkina Faso and Mali, which is investigating whether children who have received SMC, the RTS,S/AS01_E_ malaria vaccine or a combination of the two intervention up to the age of 5 years are at increased risk of uncomplicated or severe malaria in the one or two year periods after the interventions have been stopped, using a case control design. Details of the trial protocol can be found at www.isrctn.com/ISRCTN12207852.

The level of exposure to malaria in the first year of life may be especially important in the development of protective immunity to malaria as shown by a trial of chemoprophylaxis with Daraprim^R^ (pyrimethamine + dapsone) undertaken in infants in Tanzania [10]. This study showed a substantial decrease in clinical malaria and anaemia during the year in which the intervention was given but this was followed by an increased incidence of both uncomplicated and severe malaria in the subsequent year [10]. Follow up of these children until the age of four years showed that the cumulative number of episodes of uncomplicated malaria during the whole period of the study was slightly higher in children who had received the intervention than in the control children whilst the cumulative number of severe episodes in study children was lower than in the control children [11].

The probability that ‘delayed malaria’ might occur is likely to be highest following the deployment of malaria control interventions that elicit a high degree of protection during the period in which they are given but which drops off rapidly as soon as their administration is halted, as is the case for most chemopreventive measures. In contrast, vector control measures, such as ITNs, whose efficacy is lost more gradually, may allow sufficient low-density infections to occur during the period of waning protection to induce enough immunity to prevent a serious clinical outcome in the post-intervention period. Although there was initial concern that provision of ITNs to young children might lead to a shift in the peak incidence of malaria towards older children and that this might be severe, no evidence has been found that introduction of ITNs has been followed by an increase in deaths from malaria in older children [12–14] and a recent study from Tanzania has reported that the increased survival seen in children always sleeping under a net, protection achieved during the first five years of life was sustained into early adulthood [15]. Furthermore, no evidence has been found of a marked increase in the incidence of severe malaria in children aged older than five years in areas of East Africa where malaria has been effectively controlled using ITNs and other control measures and where the incidence of malaria has fallen markedly in recent years [16, 17].

The recent recommendation from the WHO SAGE committee supporting the deployment of the RTS,S/AS01_E_ [18] vaccine and the rapid progress with the R21 malaria vaccine [19], both vaccines that target young children, has raised concerns that while providing some protection against both severe and uncomplicated malaria for several years, their use may impair the development of naturally acquired immunity so that vaccinated children become at increased risk of severe malaria during the period after the vaccine induced immunity has waned [20]. During the initial follow-up period of the phase 3 trial of RTS,S/AS01_E_ vaccine (3–4 years), an increase in cases of cerebral malaria in children aged 5–17 months who received three doses of vaccine compared to the controls was seen [21], but numbers were small and an increase in cerebral malaria has not been seen in the large pilot implementation study being conducted in Ghana, Kenya and Malawi. At one site (Nanoro, Burkina Faso) which participated in the RTS,S/AS01_E_ phase 3 trial where children were followed for up to seven years, there was a significant increase in the incidence of uncomplicated malaria in children who had received three or four doses of RTS,S/AS01_E_ compared to the controls but this was not the case for severe malaria [22]. The risk of delayed malaria is likely to be less with vaccines whose efficacy is lost gradually over a period of time than is the case for chemopreventive strategies with an abrupt loss of protection.

The occurrence of ‘delayed malaria’ is not an inevitable consequence of providing effective malaria control to young children. For example, in a study in which dihydroartemisinin-piperaquine was given every four weeks or every 12 weeks to Ugandan children from the age of eight weeks to twenty-four months, children who received the intervention every four weeks had significantly fewer episodes of malaria than did children who received the intervention every twelve weeks during both the two years of the intervention and in the following year [23]. In an earlier study in Tanzania in which intermittent preventive treatment with sulfadoxine-pyrimethamine was given to infants, protection also persisted into the year after drug administration was discontinued [24]. However, as described above, in the same Tanzanian community, weekly chemoprophylaxis with pyrimethamine/dapsone in infants was followed by a subsequent increase in the incidence of malaria in the year after the intervention was stopped [10]. Whether or not ‘delayed malaria’ occurs will depend upon a number of variables including the efficacy of the intervention, its duration, the rate at which natural immunity is reacquired and, most importantly, whether there has been an overall reduction in the level of malaria transmission in the local community during the period of the intervention. The relative importance of each of these variables is not fully understood. Thus, it is important that as increasingly effective methods of controlling malaria in young children resident in highly endemic areas become available and are deployed, it is important that the longer-term impact of these interventions on the epidemiology of malaria in the study population is monitored carefully.

## Conclusions

In recent years, the the term ‘rebound malaria’ has been used to describe a variety of situations in which there has been a recurrence of malaria on withdrawal of an effective intervention or combination of interventions. In this paper, it is suggested that it is useful to differentiate between ‘resurgent malaria’, resulting from a malaria control programme applied at a population level which is not sustained, and ‘delayed malaria’ which may follow administration of highly effective interventions to young children which are not sustained beyond a specific age, even though both result from interference with development of naturally acquired immunity (Table 1). Many studies have shown that it is possible to produce a marked reduction in malaria morbidity and mortality using effective malaria control measures, even in areas of high malaria transmission, but that unless the vector population has been greatly reduced or permanently eliminated, a resurgence in malaria infection is likely to occur. However, the impact of this loss of naturally acquired immunity can be mitigated through improvements in access to effective health care after the interventions are withdrawn.

**Table 1:** Characteristics of ‘resurgent malaria’ and ‘delayed malaria’

	Resurgent malaria	Delayed malaria
Population exposed to the intervention	Usually the whole population	Usually young children
Malaria immune status on cessation of the intervention	Varied	Low
Impact of withdrawal of the intervention	An increase in incidence of malaria in the whole at risk population	An increase in incidence of malaria in older children

Current, limited evidence suggests that there may be an increase in malaria in young children protected from malaria during the period after the intervention is withdrawn. Nevertheless, all the studies done so far have shown that the benefits of the intervention far outweigh this risk, and concerns about possible ‘delayed malaria’ should not, therefore, inhibit the implementation of highly effective interventions in early childhood. If situations can be identified in which ‘delayed malaria’ is a potential risk, then steps can be taken to mitigate its possible impact, for example by making a child’s family aware of the risk, reminding the family of the need to be vigilant over the health of their child, increasing the awareness of school teachers on the risk of malaria in school-age children and providing children with a new ITN at the time that they cease to receive the intervention or when its efficacy may have waned and ensuring that access to effective diagnosis and treatment for potentially at risk groups is sustained.

## Data Availability

This is not applicable to this review paper.
